# Ecological predictors of family mealtime over time among overweight African American adolescent-parent dyads

**DOI:** 10.1007/s10865-025-00571-0

**Published:** 2025-05-15

**Authors:** Mary Quattlebaum, Allison M. Sweeney, Dawn K. Wilson

**Affiliations:** 1 Department of Psychology, University of South Carolina, Columbia (SC), USA; 2 Department of Biobehavioral and Nursing Science, College of Nursing, University of South Carolina, Columbia (SC), USA

**Keywords:** Family mealtime, Ecological, African American, Adolescent

## Abstract

African American adolescents are at risk of living in obesogenic environments, which may contribute to low-quality dietary intake and associated obesity risk. Family mealtime builds capacity for health behaviors; however, limited studies have assessed facilitators or barriers of family mealtime using an ecological approach among African American families. This study longitudinally (baseline, 8 weeks, 16 weeks) evaluated a range of ecological factors as predictors of family mealtime frequency and quality among 151 overweight African American adolescent-parent dyads (adolescent *M*age = 12.9 ± 1.7; *M*BMI%=96.3 ± 4.4; %female = 60.9% [adolescent], 96.0% [parent]) that participated in the Families Improving Together (FIT) for Weight Loss trial. Multilevel model analyses demonstrated a significant two-way interaction between perceived neighborhood healthy food access and time (*B* = 0.11, *SE* = 0.05, *p* = 0.025), such that greater perception of neighborhood access to healthy foods was associated with increased family mealtime quality over time. Further, a significant two-way interaction between family social support for healthy eating and time (*B* = 0.13, *SE* = 0.06, *p* = 0.018), such that higher levels of reported social support were associated with increased family mealtime quality over time. Finally, a marginal two-way interaction between parental limit-setting on health behaviors and time (*B* = 0.19, *SE* = 0.10, *p* = 0.069), such that greater limit-setting was associated with increased family mealtime frequency over time. These findings indicate the importance of environmental and interpersonal support in supporting family mealtime frequency and quality in African American families.

**Trial registration** ClinicalTrials.gov # NCT01796067. The trial was registered on February 21, 2013, and the first participant was enrolled July 12, 2013.

## Background

African American adolescents are at heightened risk for low-quality dietary intake (fewer fruits and vegetables, greater sweetened beverage and fat intake) and associated long-term health consequences (e.g., obesity) compared to their White peers (Merlo et al., 2020). Well-established literature, including systematic reviews, demonstrate that family mealtime is associated with increased fruit and vegetable intake, as well as lower fast food and sweetened beverage intake among adolescents (Neumark-Sztainer et al., 2010; Snuggs & Harvey, 2023). Understanding how to foster high quality family mealtime, as well as frequent family mealtime, is critical for promoting such positive health outcomes (Dallacker et al., 2018). Identifying facilitators of family mealtime frequency and quality among African American adolescents and parents is particularly important, given that African American families tend to have less frequent family mealtime compared to their non-minority counterparts (Hennessy et al., 2015; Neumark-Sztainer et al., 2003; Surjadi et al., 2017) and limited existing studies on family mealtime have focused on predominantly African American family systems.

Considering that family meals are embedded within the family context and home environment, the majority of prior research assessing facilitators of family mealtime have targeted family-level interpersonal factors (e.g., parental encouragement, parental monitoring of health behaviors, positive parenting) (Berge et al., 2010; Snuggs & Harvey, 2023). In accordance with Family Systems Theory, supportive, nurturing family interactions promote healthy adolescent behaviors, including nutritious eating behaviors (Broderick, 1993; Kitzman-Ulrich et al., 2010a, b; Wilson et al., 2017). This framework is particularly useful in consideration of family mealtime, as these meals can be a source of family routines, as well as a time for family communication focused on eating behaviors and family relationships, which may build family resilience (Masten, 2018). In fact, a recent cross-sectional study including overweight and obese African American adolescents and parents found that parental feeding practices of monitoring, reasoning/teaching, modeling healthy eating, and parent-reported authoritative parenting styles were positively associated with family mealtime frequency (Ardakani et al., 2023). Fewer studies have examined parental feeding practices of monitoring and limit-setting of eating habits, as most studies have examined adolescent- and parent-reported family encouragement for healthy eating and positive family functioning, constructs that have been positively associated with family mealtime frequency cross-sectionally (Berge et al., 2013; Poulos et al., 2014). A longitudinal study by Wilson and colleagues (2021) has demonstrated that adolescent-reported authoritative (nurturing) parenting styles moderated treatment effects for improving family mealtime frequency over 16 weeks within a sample of overweight African American adolescents. Specifically, significant three-way interactions showed that in the treatment group, increased responsiveness and lower demandingness was associated with increased family mealtime frequency over time. All together, these studies suggest that supportive family-based interpersonal support perceived by both adolescents and parents are critical facilitators of family mealtime frequency. However, limited research has examined whether parental practices of monitoring and limit-setting are predictive of family mealtime frequency and quality, and more specifically if these factors are facilitators longitudinally, which may be important for improving developmental trajectories in youth.

Prior studies have also focused on individual-level factors within adolescent samples to examine determinants of family mealtime using Bandura’s theory of self-efficacy (1986) and Ryan and Deci’s theory of motivation (2000). Understanding the role of self-efficacy and motivation in adolescence is particularly critical, as this is a developmental stage in which adolescents develop more independence and skills that set the stage for healthy behaviors into young adulthood (Keshavarz & Mounts, 2017). In line with Bandura’s work (1986), literature has shown that adolescent self-efficacy for healthy eating, self-efficacy for cooking, and adolescent involvement in family food preparation has a demonstrated link to greater family mealtime frequency (Berge et al., 2016; Woodruff & Hanning, 2009; Woodruff & Kirby, 2013). Consistent with Ryan and Deci’s work (2000), adolescent autonomous motivation for healthy eating has been linked with greater intake of healthy foods and beverages (increased fruit and vegetables, fewer junk foods and sweetened beverages) (Contento et al., 2010; Zhang et al., 2020) and may be an important predictor of family mealtime but this has not been the focus of previous studies. Importantly, cross-sectional studies have demonstrated that teen’s value of or attitudes—which are strongly tied to motivation—toward family mealtime is positively associated with family mealtime frequency (Hennessy et al., 2015; Woodruff & Kirby, 2013). These findings suggest the importance of adolescent intrapersonal-level characteristics of self-efficacy in regard to family mealtime with prior cross-sectional data, however, there are notable literature gaps on the role of adolescent motivation (intrinsic and autonomous motivation) for healthy behaviors in relation to family mealtime.

Environmental supports (e.g., home, neighborhood) may also be an important determinant of quality and frequency of family mealtime. Much of the existing literature has examined home-based environmental determinants (e.g., availability of foods in the home) of family mealtime with few studies considering broader community and neighborhood (e.g., access to markets with fresh produce) factors. Specifically, existing literature has shown that access to healthy foods in the home, including fruits and vegetables, is linked to more frequent family mealtime (Baltaci et al., 2023; Hennessy et al., 2015; Newman et al., 2015). Studies have also shown that other home-based factors, including the number of adults at the meal and the location of the meal in the home, can increase the odds of lower quality family mealtime, specifically watching television during family mealtime (Trofholz et al., 2019). Notably, a study examining neighborhood-level predictors of meal preparation at home conducted with a low-income African American sample found a positive association between shopping for food at farmers markets and grocery stores and frequency of home meal preparation (Garcia et al., 2018). Given the relationship between preparing meals at home and engaging in family mealtime (Kornides et al., 2014), neighborhood level factors, such as access to farmers markets and grocery stores, may be relevant predictors of family mealtime. Additional research is needed to fill existing gaps in the literature to better understand neighborhood level determinants of family mealtime using longitudinal study designs among African American families.

Although these prior studies are informative, they are limited in that they do not comprehensively consider the interaction of these interpersonal and environmental factors. This highlights a need to utilize a socioecological lens to fully conceptualize determinants of family mealtime. Specifically, the socioecological model purports that health behaviors are influenced by multiple factors, including perceptions of intrapersonal, interpersonal (e.g., families, friends), and community level (e.g., neighborhood) support or resources (Elder et al., 2007). To our knowledge, no prior study has utilized an ecological approach to examine determinants of family mealtime across time with African American families. Thus, the purpose of the current study was to evaluate whether various protective intrapersonal (self-efficacy for diet, motivation for diet), interpersonal (family social support for diet, family health communication, parental limit-setting, parental tracking) and environmental supports (home and neighborhood food access and availability) were predictors of family mealtime frequency and quality over time in a sample of overweight African American adolescent-parent dyads. Consistent with the Socioecological model, Motivational theory, and Family Systems Theory, we hypothesized that adolescent- and parent-reported autonomy-supportive, self-efficacious intrapersonal factors, supportive, nurturing interpersonal factors, and protective environmental-related factors would be positively associated with increased family mealtime frequency and quality among African American families using a longitudinal study design.

## Methods

### Study design

The current study analyzed secondary data obtained from the Families Improving Together (FIT) for Weight Loss trial (Wilson et al., 2015, 2022). The purpose of the FIT trial was to evaluate the efficacy of a motivational plus family-based weight-loss (M + FWL) intervention versus a comprehensive health education program on reducing body mass index (BMI) and improving diet and physical activity in overweight and obese African American adolescents and their parent (ClinicalTrials.gov ID#: NCT01796067). The FIT trial was approved by the University of South Carolina Institutional Review Board. Phase 1 of the trial tested the efficacy of an 8-week face-to-face group-based M + FWL intervention program (vs. comprehensive health education) on reducing BMI and improving diet and physical activity in overweight African American adolescents and their parent. In phase 2 of the trial, participants were re-randomized to either an 8-week tailored online intervention or a control online program resulting in a 2 (M + FWL vs. comprehensive health education group) x 2 (intervention vs. control online program) factorial design to evaluate the additive effects of the intervention modality. Full details regarding study design and procedures for the FIT trial are available (Wilson et al., 2015, 2022). The current study is longitudinal and utilizes psychosocial data from adolescents and their parent gathered at three time points including baseline, post-group intervention (8 weeks), and post-online intervention (16 weeks). The FIT Trial was guided by Self-Determination Theory, Family Systems Theory, Social Cognitive Theory, and cultural tailoring. The intervention targeted positive parenting skills, autonomy support, and cultivating self-efficacy by building behavioral skills, such as goal setting and monitoring. The current study included families in the intervention and comprehensive health education programs. Analyses controlled for treatment condition for the group program and online program, given that the intervention did not directly target family meals as an outcome.

### Participants

The current study included 151 African American parents and adolescents that participated in the FIT trial (Wilson et al., 2015, 2022). FIT participants were recruited between 2013 and 2018 and follow-up measures for the final cohort concluded in Spring of 2019. Adolescents were eligible for participation if they (1) identified as African American, (2) were overweight or obese (BMI ≥ 85th percentile), (3) were between 11 and 16 years old, (4) had internet access, and (5) had a parent willing to participate in the study. Adolescents were excluded from the study if they had a medical or psychiatric condition that might interfere with physical activity or dietary habits, were taking any medications that could impact their weight or appetite, or if they were currently enrolled in another structured weight-loss program. If more than 1 parent was interested in participating, the family determined which parent would participate and the other parent could receive access to the FIT program’s workbook pages. Participants residing in Columbia, SC, were recruited through local clinics, schools, and community centers (i.e., churches and recreational centers) using culturally relevant recruitment approaches (Huffman et al., 2016). Culturally relevant recruitment approaches included culturally-relevant ads (African American publications), community partners that serve African American families (Boys and Girls Club), and sociocultural events such as Black History Month community events and events at African American churches. Adolescents and their parents provided written informed assent and consent, respectively, prior to participation in the study.

### Procedures

Measures were collected from FIT families at baseline, following the face-to-face program (8 weeks), following the online program (16 weeks), and at 6-months post intervention. The current study only examined data from baseline, 8 weeks, and 16 weeks. Baseline assessments for the FIT trial were completed over a 2-week orientation period before starting the intervention. At baseline, 8 weeks, and 16 weeks, parent-adolescent dyads completed anthropometric measurements (weight, height) and psychosocial surveys (demographic variables, family mealtime, intrapersonal, interpersonal, and environmental variables). Study measures were collected by certified measurement staff that were blind to group assignment. Participants were compensated with $110 for their participation in the intervention and measurements across all timepoints.

### Measures

#### Demographics and anthropometrics

Demographics were collected on participating families and included age, sex, race, parent annual household income, parent education, parent marital status, and number of children in the home. To measure race, participants were asked to identify which of the following best describes their race: Black or African American, Asian, Middle Eastern, American Indian or Alaskan Native, Native Hawaiian, Other Pacific Islander, White, or Other. Adolescent participants were also asked to confirm if they had 3 African American grandparents. Parents reported annual household income by selecting one of the following categories: Less than $10,000, $10,000 to $24,999, $25,000 to $39,999, $40,000-$54,999, $55,000 to $69,999, $70,000 to $84,999, or $85,000 or more. Parents reported on their highest level of education completed by selecting one of these categories: Never attended school or only attended kindergarten, Grades 1–8, Grades 9–11, Grade 12 or GED, College 1–3 years, College 4 years or more, or Graduate training or professional degree. Parent marital status was measured using the following categories: Married, Separated, Divorced, Widowed, Never Married, or In an unmarried couple. Adolescent and parent height and weight were measured for eligibility by a trained research assistant with a Shorr height board and SECA 880 digital scale, respectively. Height was measured in centimeters and weight was measured in kilograms were taken twice. BMI was calculated with an average of the height and weight measurements based on the Center for Disease Control (CDC) for adolescents and adults, respectively.

### Intrapersonal predictors

#### Self-efficacy for a healthy diet (parent and adolescent-reported)

Adolescent and parent self-efficacy for eating a healthy diet was measured using an adapted version of the Self-Efficacy for Eating Habits Scale (Sallis et al., 1988a, b). The modified scale has previously demonstrated predictive validity to specifically evaluate self-efficacy for healthy eating in African American adolescents (Wilson et al., 2002). This self-report 10-item measure integrates relapse prevention and behavioral skills. Respondents are asked to rate how confident they are that they could continue to eat nutritiously for at least six months when experiencing specific challenges. Sample items include “How sure are you that you can stick to eating healthfully when eating with family?” Responses are scored on a 5-point Likert scale that ranges from ‘1 = I know I cannot’ to ‘5 = I know I can.’ Sallis et al. (1988a, b) demonstrated adequate internal consistency (ranging from α = 0.85 to 0.93), and previous studies conducted with African American youth have demonstrated adequate reliability and validity of the instrument (Wilson et al., 2005). Prior research with underserved youth has also shown the scale to be predictive of fruit and vegetable intake (Wilson et al., 2002, 2005).

#### Intrinsic motivation for a healthy diet (adolescent-reported)

Adolescents completed an 8-item measure that assessed their intrinsic motivation for eating a healthy diet. This measure was developed by Wilson and colleagues (2002) and was adapted to improve psychometric properties (Lawman et al., 2011, 2012; St. George et al., 2013). Sample items include “Eating healthy is important to me,” and “I am excited about eating healthy on most days.” Responses are scored on a 6-point Likert scale ranging from ‘1 = Strongly disagree’ to ‘6 = Strongly agree.’ Reliability estimates for the adapted scale has ranged from 0.82 to 0.88 (Lawman et al., 2011, 2012; St. George et al., 2013). Furthermore, construct validity of the scale has been previously established through significant associations with dietary outcomes in primarily African American adolescents (Wilson et al., 2002).

#### Autonomous motivation for a healthy diet (parent and adolescent-reported)

Adolescents and parents reported their autonomous motivation for healthy eating using the Treatment Self-Regulation Questionnaire (TSRQ), which is a 15-item measure (Ryan & Connell, 1989). Participants reported on various reasons they would eat a healthy diet. Sample items include “Because it is an important choice I really want to make,” “Because I feel pressure from others to do so.” Responses were scored on a 7-point Likert scale that ranges from ‘1 = Not at all true’ to ‘7 = Very true.’ Previous work with predominantly African American youth samples have indicated adequate predictive validity and adequate concurrent validity of the scale (Denman et al., 2016; Levesque et al., 2007).

### Interpersonal predictors

#### Social support for diet (adolescent-reported)

Adolescent-reported support from family for healthy eating was assessed using the modified version of the Support for Diet and Exercise Behaviors Scales (Sallis et al., 1987). In line with prior modifications made to this scale (Peterson et al., 2013), negatively worded items were removed, and positive worded items were used to assess adolescent perceptions of support for diet from family. Previous studies have shown that when negative items are reverse coded, they may introduce a method bias (Lawman et al., 2011). Adolescents were asked to report on the frequency with which they have received social support regarding their healthy eating habits from a family member in their household. Adolescents were instructed to base their responses for this 6-item scale off of the past month. Sample items include “In the past month, my family (or members of my household) complimented me on my eating habits (“Keep it up” “We are proud of you”).” Responses were scored on a 5-point Likert scale that ranged from ‘1 = None’ to ‘5 = Very often.’ Construct validity has been supported in prior studies using this scale with youth (Kitzman-Ulrich et al., 2010a, b; Sallis et al., 1988a, b, 1999).

#### Family health communication (adolescent-reported)

Adolescents completed an adapted version of the Health Care Climate Questionnaire, which assesses adolescents’ attitudes regarding conversations with their parents about weight, diet, and exercise (Williams et al., 1996, 1998). This adapted 9-item scale includes the following sample items, “When it comes to weight, diet, and exercise, my parent listens to me,” and “When it comes to weight, diet, and exercise, my parent is too pushy.” Negatively worded items are reverse coded. Responses are scored on a 4-point Likert scale ranging from ‘1 = Not at all’ to ‘4 = A lot.’

#### Limit-setting and tracking of adolescent’s health behaviors (parent-reported)

Parents completed the Parenting Strategies for Eating and Activity Scale (PEAS), which is a 13-item scale that measures parental limit-setting and tracking of adolescent’s health behaviors (Arredondo et al., 2006; Larios et al., 2009). Adolescent health behaviors assessed with this measure include screen time, sedentary behavior, sweetened-beverage intake, physical activity, fruit/vegetable intake, salty snack food intake, etc. Sample items of limit-setting include “I limit the amount of snacks my child eats” and sample items of tracking include “How often do you keep track of the high fat foods your child eats?” Responses are scored on a 5-point Likert scale that ranges from ‘0 = Never’ to ‘4 = Always.’ Limit-setting and tracking were scored as distinct subscales. Construct validity of the PEAS has previously been supported through comparisons of the PEAS with the Child Feeding Questionnaire (Birch et al., 2001) among a sample of ethnically diverse parents (Larios et al., 2009).

### Environmental predictors

#### Food access and availability in home (parent and adolescent-reported)

Adolescents and parents were asked how often healthy and unhealthy food items were available within their home environment utilizing a 17-item scale (Ding et al., 2012). Examples of healthy food items included raw fruit and vegetables, 100% fruit juice, and unsweetened breakfast cereal; examples of unhealthy food items encompassed chips, candy, sodas, and sweetened breakfast cereals. Responses were scored on a 5-point Likert scale ranging from ‘1 = Never’ to ‘5 = Always.’ Access to healthy foods and unhealthy foods in the home were scored as distinct subscales.

#### Perceptions of neighborhood food access (parent-reported)

Parents reported on their perception of their neighborhood’s access to healthy foods using a modified scale (Barnes et al., 2019). The instructions for the 7-item scale specified that one’s neighborhood should be conceptualized as the area within a 20-minute walk or approximately 1 mile from their home. Sample statements included “A large selection of fresh fruits and vegetables is available in my neighborhood,” “In my neighborhood, it is easy to buy healthy foods,” and “The fresh fruits and vegetables in my neighborhood are of high quality.” Responses were scored on a 5-point Likert scale that ranged from ‘1 = Strongly Agree’ to ‘5 = Strongly Disagree.’ Scores were reverse coded to simplify the interpretation of the scores.

### Outcome variables

#### Family mealtime frequency & quality (parent and adolescent-reported)

Adolescents and parents were asked the frequency and quality of their family mealtimes during a typical week using a validated scale (Neumark-Sztainer et al., 2010). This scale has been used in diverse racial populations and shown to have construct validity and reliability (α = 0.73) (Neumark-Sztainer et al., 2010). To assess family mealtime frequency, respondents were asked a single item: “In a typical week, how many times did all or most of your family living in your house eat a meal together at home?” The responses ranged from ‘1 = Never’ to ‘6 = More than 7 times.’ Adolescents and parents were also asked to rate the quality of their family mealtimes during a typical week using a 4-item scale. Family mealtime quality was assessed with sample items that include “I enjoy eating meals with my family,” and “In my family, mealtime is a time for talking with other family members.” Responses were scored on a 4-point Likert scale that ranged from ‘1 = Strongly disagree’ to ‘4 = Strongly agree.’ Family mealtime frequency and quality were scored as distinct subscales.

### Analysis plan

Multilevel growth modeling analyses were conducted using R software with the nlme package. Relevant assumptions, including the presence of influential cases, normality, and heteroscedasticity, were evaluated and indicated no unexpected findings. Likelihood ratio tests were run to assess the best fitting model, which indicated that the best fitting model was a linear growth model with a fixed effect for time and random effect for treatment group. This modeling approach was used for all subsequent analyses and is consistent with the approach used in prior Project FIT analyses (Wilson et al., 2022). Next, using a hierarchical approach, a series of chi-square difference tests compared a covariate only model, the addition of main effects, and the addition of interaction terms. Across all models, the likelihood ratio tests revealed that the best model included covariates, main effects, and two-way interaction terms (*p* < 0.05). Covariates included group and online randomization, adolescent age, adolescent sex, yearly income, and time (0 = baseline, 1 = 8 weeks, 2 = 16 weeks). Adolescent age was mean centered, while adolescent sex was dummy coded (1 = male, 0 = female). All predictor variables were calculated by norming each item before summation to allow each item to contribute equally to the overall scale score. Summed scale scores were transformed to z scores to aid in analysis and interpretation of statistical models. The models tested a series of two-way interactions between time and the predictor variables when predicting family mealtime outcomes. A series of four models were conducted: (1) family mealtime quality with parent-reported variables; (2) family mealtime quality with adolescent-reported variables; (3) family mealtime frequency with parent-reported variables; and (4) family mealtime frequency with adolescent-reported variables.

The current study included 151 adolescent-parent dyads from the full FIT sample (*N* = 241 adolescent-parent dyads). Psychosocial data were missing from < 2% of adolescents and parents at baseline and < 37% of adolescents (36.7%) and parents (29.9%) at 16-weeks due to participant attrition over time. Multiple imputations were used to address the limited (< 2%) missing baseline data of the outcomes of the current study (family mealtime quality, family mealtime frequency) using the MICE package in R. The imputations were conducted to minimize the likelihood of biased estimates and meet missing at random assumptions. Given that the primary outcomes of the FIT trial were physical activity and weight status (Wilson et al., 2022), multiple imputations were not conducted with the psychosocial predictor measures included in the current study. Multilevel growth modeling analyses with nlme were used in the current study, and thus analyses included all available data, and the model implicitly weighted the results based on what data was available. To determine if the current study sample was generalizable to the larger full FIT sample, we assessed for potential differences in sample demographics and outcome measures (Table 1). Overall, there were similar demographics between the current study sample and full sample. The current study sample has slightly fewer females (60.9% vs. 64.0%) and slightly lower education levels than the larger sample (20.5% with 12 years or fewer vs. 15.8%). Otherwise, the current study sample and full sample were largely homogenous.

**Table 1:** Baseline characteristics

	Current sample (*N* = 151 dyads)	Full Sample (*N* = 241 dyads)
Adolescent age (years), M ± SD	12.9 ± 1.7	12.83 ± 1.75
Parent age (years), M ± SD	43.6 ± 8.96	43.2 ± 8.65
Adolescent sex (female, %)	60.9	64.0
Parent sex (female, %)	96.0	96.0
Adolescent BMI Percentile (kg/m^2^%), M ± SD	96.3 ± 4.4	96.61 ± 4.25
Parent BMI (kg/m^2^), M ± SD	37.90 ± 8.76	37.75 ± 8.79
Annual Household Income, %		
Less than 10k	17.2	14.9
10–24k	20.5	20.3
25–39k	27.2	26.6
40–54k	12.6	12.9
55–69k	7.9	8.7
70–84k	4.0	5.0
85k+	10.6	10.0
Parent Education, %		
9–11 years	3.3	2.5
12 years	17.2	13.3
Some college	33.8	41.1
4-year college	21.2	18.7
Professional	24.5	22.8
Parent Marital Status, %		
Married	35.8	34.4
Number of Children at Home, M ± SD	2.07 ± 1.18	2.05 ± 1.20
Adolescent-report FM Quality, M ± SD	3.13 ± 0.74	3.14 ± 0.77
Parent-report FM Quality, M ± SD	3.58 ± 0.55	3.60 ± 0.58
Adolescent-report FM Frequency, M ± SD	3.44 ± 1.58	3.46 ± 1.62
Parent-report FM Frequency, M ± SD	3.36 ± 1.41	3.54 ± 1.50

Note: M = mean; SD = standard deviation; BMI = body mass index, kg/m^2^ = kilograms per meter squared; k = thousand; FM = family mealtime. Family Mealtime frequency ranges from 1 = Never to 6 = More than 7 times (weekly). Family Mealtime quality scores range from 1 to 4

## Results

### Demographics

Table 1 outlines the descriptive data from the current sample and the larger FIT sample. The current study sample included 151 African American parent-adolescent dyads. Adolescent and parent participants were primarily female (adolescent: 60.9%; parent: 96.0%). The mean age for adolescents was 12.9 (*SD* = 1.7) and the mean BMI percentile was 96.3 (SD = 4.4). Of note, the majority of families reported annual household income as below $40,000 (64.9%) and 64.2% of parents were not married. Analyses were run to determine if family mealtime frequency or quality was significantly different based on if parents were married or not married; findings showed no significant differences. The mean baseline family mealtime frequency on a 6-point scale was 3.44 (SD = 1.58) for adolescent-report and 3.36 (SD = 1.41) for parent-report and the mean family mealtime quality on a 4-point scale was 3.13 (SD = 0.74) for adolescent-report and 3.58 (SD = 0.55) for parent-report.

### Correlational analyses

Bivariate correlational analyses were performed with demographic variables, predictor variables, and family mealtime frequency and quality at baseline (Table 2). Adolescent social support for healthy eating was significantly associated with adolescent family health communication (*r* = 0.456), adolescent-reported healthy food home access (*r* = 0.233), adolescent-reported unhealthy food home access (*r* = 0.204), adolescent-reported family mealtime frequency (*r* = 0.180) and adolescent-reported family mealtime quality (*r* = 0.274). Adolescent-reported family mealtime quality was positively correlated with family social support for healthy eating (*r* = 0.274), family health communication (*r* = 0.419), and access to healthy (*r* = 0.187) and unhealthy (*r* = 0.210) foods in the home. For both parents and adolescents, family mealtime frequency was positively correlated with family mealtime quality (parents: *r* = 0.232; adolescents: *r* = 0.351).

**Table 2:** Correlations among demographics, predictor variables, and family mealtime

	1	2	3	4	5	6	7	8	9	10	11	12	13	14
1. Adolescent Male	**-**													
2. Adolescent Age	-0.029	**-**												
3. Parent Income	-0.070	-0.035	**-**											
4. Social Support	0.023	-0.171*		**-**										
5. Neighborhood Access	0.109	0.095	0.173*	0.108	-									
6. Family Health Communication	0.032	-0.189*	0.008	0.456*	0.044	-								
7. Parent Unhealthy Food in Home	0.025	-0.030	-0.065	-0.065	-0.015	-0.099	-							
8. Parent Healthy Food in Home	-0.181*	0.136	-0.008	0.001	0.142	0.124	0.026	-						
9. Parent FM Quality	-0.051	-0.075	-0.089	0.039	0.156	0.156	-0.042	0.133	-					
10. Parent FM Frequency	-0.102	-0.234*	0.033	0.041	-0.028	0.036	-0.057	0.193	0.232*	-				
11. Child Unhealthy Food in Home	0.071	-0.116	-0.141	0.204*	0.006	0.121	0.407*	0.026	0.032	-0.028	-			
12. Child Healthy Food in Home	-0.079	-0.005	-0.057	0.233*	0.047	0.065	-0.016	0.346*	-0.020	-0.001	0.436*	-		
13. Child FM Quality	0.054	-0.149	0.002	0.274*	-0.059	0.419*	-0.002	0.028	0.172*	0.151	0.210*	0.187*	-	
14. Child FM Frequency	0.053	-0.169*	0.068	0.180*	-0.044	0.102	-0.075	0.031	0.103	0.449*	0.118	0.098	0.351*	-

*Indicates correlations significant with alpha criteria of 0.05. Column headings correspond to row names. FM = Family Mealtime

### Family mealtime quality

#### Parent analyses

**Fig. 1:**
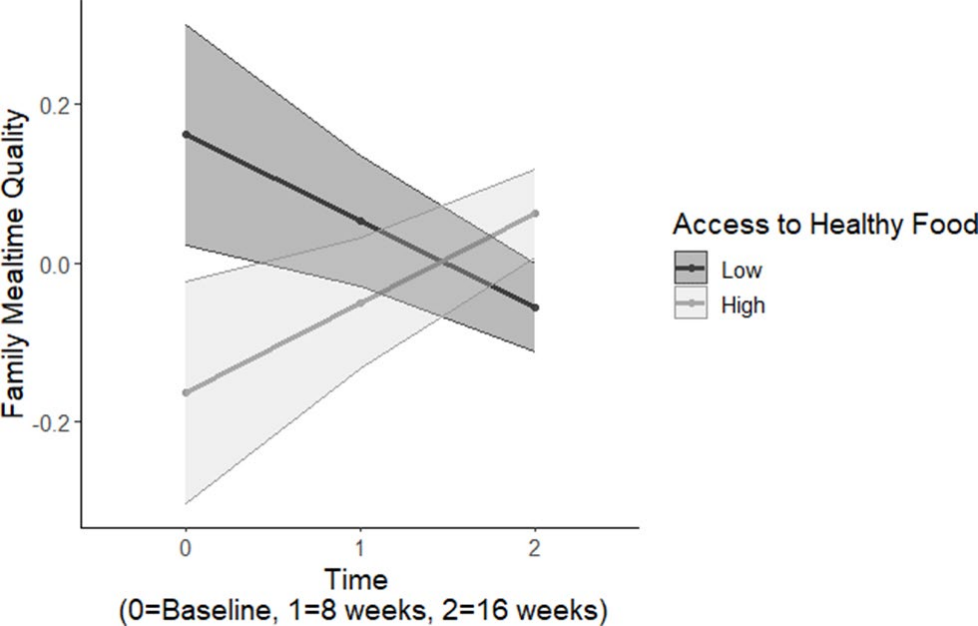
Interaction between time and perceived neighborhood access to healthy food on family mealtime quality over time

Results demonstrated a significant two-way interaction between parent-reported perceived neighborhood access to healthy foods and time on parent-reported family mealtime quality (*B* = 0.11, *SE* = 0.05, *p* = 0.025; Table 3). Simple slopes analyses demonstrated that among parents with higher reported neighborhood access to healthy foods (+ 1 SD), family mealtime quality increased over time (*B* = 0.13, *SE* = 0.07, *p* = 0.058) (see Fig. 1). However, among parents with lower reported neighborhood access to healthy foods (-1 SD), family mealtime quality decreased over time (*B* = -0.13, *SE* = 0.07, *p* = 0.065). There were no significant main effects or additional two-way interaction effects in this model.

**Table 3:** Multilevel model of parent variables predicting family mealtime quality

Model/Parameter	B	SE	t-value	*p*-value
Intercept	0.732	0.33	2.219	0.026
Group Randomization	0.092	0.083	1.111	0.272
Online Randomization	0.0005	0.078	0.006	0.995
**Child Age**	**-0.0553**	**0.022**	**-2.427**	0.016*
Child Sex	-0.004	0.080	-0.539	0.590
Income	-0.015	0.021	-0.710	0.477
Autonomous Motivation for Diet	0.079	0.113	0.695	0.487
Time	0.001	0.048	0.027	0.979
Self-Efficacy for Diet	0.054	0.118	0.455	0.649
Parental Limiting of Health Behaviors	-0.082	0.146	-0.558	0.577
Parental Tracking of Health Behaviors	0.180	0.152	1.187	0.236
Perceived Neighborhood Food Access	-0.162	0.101	-1.602	0.110
Access to unhealthy food in home	-0.012	0.108	-0.107	0.915
Access to healthy food in home	-0.060	0.110	-0.549	0.584
Time*Autonomous Motivation	0.022	0.057	0.390	0.697
Time*Self-Efficacy	-0.009	0.059	-0.149	0.882
Time*Limiting	0.091	0.073	1.250	0.212
Time*Tracking	-0.056	0.075	-0.748	0.455
Time*Neighborhood Access	**0.110**	**0.049**	**2.244**	0.025*
Time*Unhealthy Food Access	0.011	0.054	0.200	0.842
Time*Healthy Food Access	0.082	0.054	1.547	0.122

#### Adolescent analyses

**Fig. 2:**
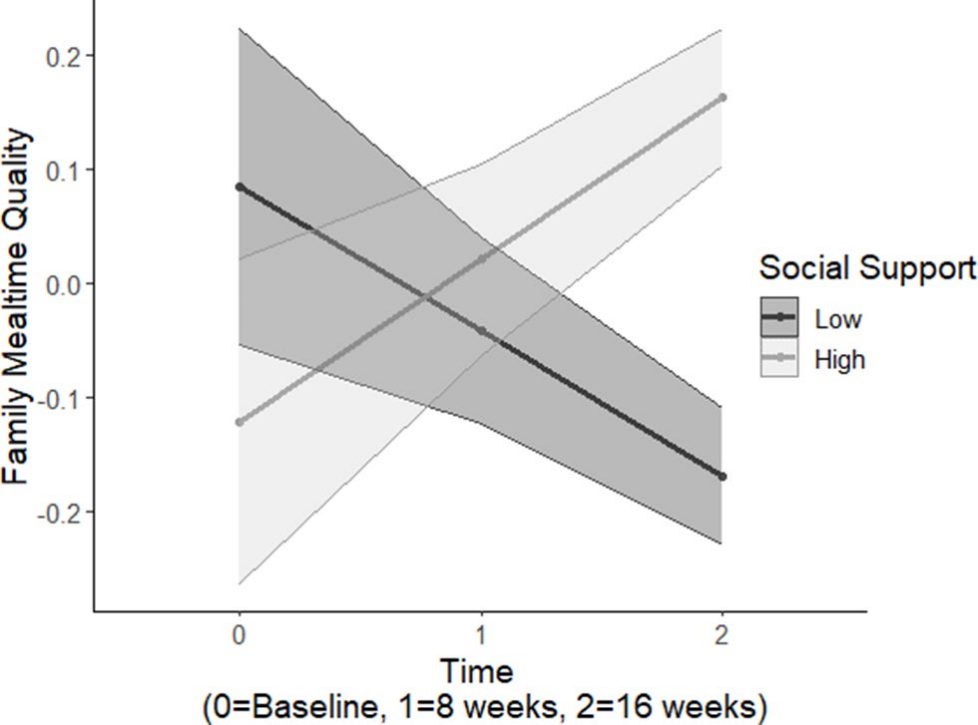
Interaction between time and social support on family mealtime quality over time

Results demonstrated a significant main effect of adolescent-reported family health communication on family mealtime quality (*B* = 0.339, *SE* = 0.100, *p* = 0.001; Table 4). Additionally, analyses demonstrated a significant two-way interaction between adolescent-reported social support for healthy eating and time (*B* = 0.134, *SE* = 0.057, *p* = 0.018) on adolescent-report family mealtime quality. Simple slopes analyses revealed that higher adolescent-reported family social support for health behaviors (+ 1 SD) was associated with greater family mealtime quality over time (*p* = 0.084), whereas lower adolescent-reported family social support for health behaviors (-1 SD) was associated with lower family mealtime quality over time (*p* = 0.126) (see Fig. 2).

**Table 4:** Multilevel model of adolescent variables predicting family mealtime quality

Model/Parameter	B	SE	t-value	*p*-value
Intercept	0.211	0.294	0.718	0.473
Group Randomization	0.085	0.082	1.042	0.303
Online Randomization	0.300	0.069	0.438	0.662
Child Age	-0.032	0.020	-1.561	0.119
Child Sex	0.114	0.073	1.553	0.121
Income	0.025	0.020	1.277	0.202
**Family Health Communication**	**0.339**	**0.100**	**3.377**	0.001*
Time	0.007	0.042	0.161	0.872
Autonomous Motivation for Diet	0.166	0.102	1.631	0.103
Self-Efficacy for Diet	0.604	0.112	0.541	0.587
Intrinsic Motivation for Diet	0.052	0.129	0.402	0.688
Social Support for Diet	-0.103	0.112	-0.918	0.359
Access to unhealthy food in home	0.120	0.103	1.159	0.247
Access to healthy food in home	0.098	0.106	0.924	0.356
Time*Family Health Communication	-0.009	0.050	-0.177	0.859
Time*Autonomous Motivation for Diet	0.009	0.053	0.165	0.869
Time*Self-Efficacy for Diet	-0.102	0.057	-1.798	0.073
Time*Intrinsic Motivation for Diet	0.027	0.067	0.398	0.691
Time*Social Support for Diet	**0.134**	**0.057**	**2.372**	0.018*
Time*Unhealthy Food Access	-0.048	0.052	-0.918	0.359
Time*Healthy Food Access	-0.006	0.054	-0.111	0.912

### Family mealtime frequency

#### Parent analyses

Results demonstrated a significant main effect of parental tracking of health behaviors on parent-reported family mealtime frequency (*B* = 0.507, *SE* = 0.216, *p* = 0.019; Table 5), such that greater parental tracking was associated with greater family mealtime frequency. No significant two-way interaction effects were found in this model; however, there was a marginal interaction between parental limit-setting on health behaviors and time (*B* = 0.189, *SE* = 0.104, *p* = 0.069) demonstrating that greater limits set on health behaviors was associated with somewhat greater family mealtime frequency across time.

**Table 5:** Parent family mealtime frequency

Model/Parameter	B	SE	t-value	*p*-value
Intercept	4.955	0.483	10.263	0.000
Group Randomization	0.081	0.140	0.578	0.567
Online Randomization	0.072	0.111	0.653	0.514
Child Age	-0.099	0.033	-3.010	0.003
Child Sex	-0.186	0.117	-1.587	0.113
Income	-0.033	0.032	0.302	0.302
Autonomous Motivation for Diet	-0.045	0.162	0.780	0.800
Time	-0.023	0.068	0.736	0.763
Self-Efficacy for Diet	0.166	0.169	0.325	0.325
Parental Limiting of Health Behaviors	-0.230	0.209	0.272	0.272
**Parental Tracking of Health Behaviors**	**0.507**	**0.216**	**2.345**	0.019*
Perceived Neighborhood Food Access	-0.098	0.144	-0.680	0.497
Access to unhealthy food in home	0.137	0.154	0.891	0.373
Access to healthy food in home	0.091	0.156	0.588	0.557
Time*Autonomous Motivation	0.004	0.081	0.048	0.962
Time*Self-Efficacy	-0.020	0.084	-0.241	0.810
Time*Limiting	0.189	0.104	1.820	0.069
Time*Tracking	-0.182	0.107	-1.710	0.088
Time*Neighborhood Access	0.092	0.070	1.302	0.193
Time*Unhealthy Food Access	-0.078	0.077	-1.013	0.312
Time*Healthy Food Access	0.043	0.076	0.568	0.571

#### Adolescent analyses

There were no significant main effects nor two-way interaction effects in this model.

## Discussion

The current study evaluated the role of ecological predictors (intrapersonal, interpersonal, environmental) on the quality and frequency of family mealtime over time among African American adolescents. As hypothesized, results showed that over time greater parent-reported perceived neighborhood access to healthy foods was associated with increased family mealtime quality. Further, greater adolescent-reported family social support for healthy eating was significantly associated with greater family mealtime quality over time. Finally, there was a significant main effect of parental tracking of health behaviors and family mealtime frequency, as well as a marginal interaction effect that showed greater parental limit-setting on health behaviors over was associated with increased family mealtime frequency over time. There was also a significant main effect for family health communication on quality of family mealtime. Taken together, these findings suggest the importance of protective environmental (perceived neighborhood access to healthy foods) and supportive interpersonal (family social support for healthy eating, parental tracking and limit-setting, family health communication) determinants in cultivating family mealtime quality and frequency over time among African American families.

The findings of the current study are particularly novel as they are the first to our knowledge to examine ecological determinants of family mealtime quality over time with African American families. While prior work has primarily focused on family mealtime frequency, the current study fills a gap in existing literature by examining determinants of both frequency and quality of family mealtime. The current study, similar to past work (Fulkerson et al., 2006), demonstrated a positive association between family mealtime frequency and quality. Understanding how to cultivate high-quality family mealtime, including a positive atmosphere and supportive communication around health behaviors, is equally as important as increasing the frequency of family mealtime to improve youth developmental trajectories.

An important finding of the current study was that perceptions of neighborhood healthy food access predicted family mealtime quality over time among African American families. Similarly, prior literature has shown that healthy food access in the home is associated with increased family mealtime frequency (Baltaci et al., 2023; Newman et al., 2015). Additional studies have shown that shopping at farmers markets and grocery stores is positively associated with preparing meals at home (Garcia et al., 2018), which has a known association with greater family mealtime frequency (Kornides et al., 2014). The current study also demonstrated a significant positive association between perceived neighborhood healthy food access and parent income, which aligns with prior work indicating that families within a higher socioeconomic status tend to have greater access to healthy foods (Neumark-Sztainer et al., 2003). There was no relationship between home food access (unhealthy or healthy food) and family mealtime found in the current study, suggesting the value in examining broader community-level predictors of family mealtime outside the home environment. Findings from our study demonstrate that protective neighborhood factors are important predictors of family mealtime quality, which is a novel addition to the literature that has largely emphasized the protective nature of home-environment on family mealtime. Future studies are needed to replicate the current study findings and further explore neighborhood-level determinants of family mealtime.

Findings from the current study also suggest that supportive-nurturing interpersonal factors related to health behaviors are important determinants of family mealtime, which is consistent with Family Systems Theory (Broderick, 1993). Prior literature has shown that positive and supportive familial practices may either directly cultivate positive health behaviors or indirectly promote positive health behaviors by means of supporting adolescent independence and mastery related to health resulting in adolescent self-efficacy and motivation (Bandura, 2004; Kitzman-Ulrich et al., 2010a, b; Lawman et al., 2011; Ryan & Deci, 2000). In the current study, results showed that greater family social support for healthy eating, including instrumental and emotional support, significantly predicted increased family mealtime quality over time. The current study also demonstrated a significant main effect of positive family health communication (e.g., listening, understanding, shared decision-making, encouragement) on family mealtime quality. These findings expand on prior work showing that parental encouragement of healthy eating (Ardakani et al., 2023; Masten, 2018) and supportive parenting practices broadly (Wilson et al., 2021) are associated with increased family mealtime frequency to demonstrate the role of family social support and positive communication related to health behaviors on the quality of family mealtime. Together, these findings suggest that supportive interpersonal factors related to health behaviors may be an important point of intervention for family-based health promotion trials.

The current study also found a marginally significant interaction effect between parental limit-setting of health behaviors and family mealtime frequency. Additionally, findings demonstrated a significant main effect of parental tracking health behaviors on increased family mealtime frequency. Consistent with these findings, prior work with a sample of African American adolescents demonstrated a positive association between parental monitoring and family mealtime frequency (Ardakani et al., 2023). While parental tracking/monitoring and limit-setting may be viewed as supportive oversight, more restrictive parental feeding practices, including parental demandingness, may be viewed by adolescents as more restrictive or controlling and may attenuate one’s autonomy or decision-making. In fact, parental demandingness has previously been associated with less frequent and lower quality family mealtime (Dallacker et al., 2019; Wilson et al., 2021). Thus, these findings extend the limited literature on parental tracking and limit-setting of health behaviors as facilitators of family mealtime and further reinforce the value of supportive interpersonal factors in predicting healthy outcomes.

Across adolescent and parent models, there were no intrapersonal factors that were significant predictors of family mealtime frequency or quality. The majority of prior work examining intrapersonal determinants of family mealtime has focused on competency and self-efficacy related to cooking (Berge et al., 2016; Woodruff & Kirby, 2013), whereas the current study examined intrapersonal constructs specific to healthy eating, including self-efficacy and motivation for healthy eating. It is plausible that self-efficacy and motivation for cooking may be more relevant in cultivating family mealtime than self-efficacy and motivation for healthy eating, given that home meal preparation has been shown to predict more frequent family mealtime (Berge et al., 2016). Prior literature has demonstrated that adolescent values regarding family mealtime have been linked to family mealtime frequency (Hennessy et al., 2015; Woodruff & Kirby, 2013). It may be beneficial for future studies to examine values and motivation specifically for family mealtime—rather than healthy eating broadly—as a predictor of family mealtime frequency and quality. Further, given the role of parent-adolescent reciprocal interactions in cultivating self-efficacy and motivation around health behaviors, additional work is needed to elucidate the influence of intrapersonal factors across a variety of interpersonal or community-level contexts over time.

Despite the novelty of the current study, there are limitations to consider when interpreting the findings. Participants for the current study were recruited between 2013 and 2018 and 6-month follow-up measures for the final cohort concluded in Spring of 2019; thus, the current data is slightly outdated. However, as described above, our findings align with studies that collected data in more recent years and thus we argue that our findings are likely salient to date. The current study sample includes an underrepresented population of predominantly low-income overweight/obese African American younger adolescents and predominantly non-married (64.2%) mothers from South Carolina. Of note, existing literature has shown that younger adolescents tend to have greater frequency of family meals than older adolescents (Snuggs & Harvey, 2023); and thus, our younger sample of adolescents may have had greater rates of family meals than an older sample of African American adolescents. Conversely, literature on family meals has historically shown lower rates of family meals among lower income, single-parent households (Snuggs & Harvey, 2023). Upon further analysis, we found no differences in family mealtime frequency or quality based on if parents in the current study were married or not married. The current study assessed for marital status; however, we did not assess whether other adults or guardians (e.g., grandparents, non-married partner) were living in the home. This may be important for future studies to provide insight on who is present during family meals. Although the current study sample may have minimal variability or generalizability beyond this population, this study fills a gap in family mealtime literature by including an often understudied and higher risk population of African American families. Moreover, due to attrition over the course of the intervention (baseline, 8-weeks, 16-weeks), the current sample is smaller than the larger FIT trial sample size. Analyses used all available data and the model implicitly weighted the results based on what data was available; additionally, the baseline family mealtime data of the current study were imputed to address the minimal missing data. The demographic and outcome variables in the current study sample and the larger FIT sample were compared for generalizability and were similar, indicating that our findings would likely generalize to the larger FIT sample. Nonetheless, additional studies are needed to replicate these findings in larger sample sizes.

## Conclusions

To our knowledge, the current study is the first to examine various ecological predictors of family mealtime quality and frequency among overweight African American adolescents and parents. Findings underscore the importance of family social support, parental tracking and limit-setting, family health communication, and neighborhood healthy food access for promoting family mealtime frequency and quality among African American adolescents and families. Given the literature gap in examining how to cultivate both high quality and frequent family mealtime, there is a need for further research to expand on these interpersonal and neighborhood-level factors with African American families across various contexts.

## Data Availability

The datasets used and/or analyzed during the current study are available from the corresponding author on reasonable request.
